# Fancies and Fallacies of Spatial Sampling With Transcranial Magnetic Stimulation (TMS)

**DOI:** 10.3389/fpsyg.2018.01171

**Published:** 2018-07-05

**Authors:** Luigi Cattaneo

**Affiliations:** Dipartimento di Neuroscienze, Biomedicina e Movimento, University of Verona, Verona, Italy

**Keywords:** magnetic stimulation, non-invasive brain stimulation, aliasing, anti-aliasing, Nyquist, spatial resolutions, sampling frequency, brain mapping

## TMS as a brain-mapping tool

Transcranial magnetic stimulation (TMS) is a procedure by which an electrical field is induced in the brain. The ensuing extracellular electrical current determines supra-threshold depolarization of the neuronal membrane, ultimately resulting in action potentials. Inductor coils with appropriate shapes produce peaks in the induced magnetic field that result in induced electrical fields that are referred to as “focal” because their size is over two magnitudes smaller than the cortical surface that potentially could be sampled. Focal TMS is commonly used in cognitive neuroscience as a brain mapping tool, though certain applications of TMS escape the brain-mapping logic, for example, manipulations with repetitive TMS in the domain of cortical rhythms, TMS used to produce a measurable cortical output (such as MEPs or phosphenes) or the use of TMS in state-dependent paradigms. These experimental strategies will not be the object of discussion here. The focal electrical field of TMS in brain mapping is used as a probe for sampling the 2-dimensional space of the cortical surface. TMS does not produce data by itself but only if coupled with behavioral or physiological measures. Therefore, TMS experiments describe the spatial distribution of the effects of focal stimulation on any behavioral measure that has been chosen. Consequently, in cognitive neuroscience, TMS results are commonly described in cartographic terms: “cortical area x is necessary for behavioral function y.” In my opinion, there are several caveats to be considered before similar inference in the spatial domain can be drawn. As any functional brain-mapping procedure, TMS-brain mapping is a process of spatial sampling and reconstruction, similar to those employed in many communication systems as digital photography or video broadcasting (Dubois, 1985). The brain is the (analogic) native image, which is sampled, obtaining an array of points, which are then used to reconstruct the original image (the brain map and, ultimately, our model of how the brain works). An efficient sampling-reconstruction procedure produces a reconstructed image that is as similar as possible to the native image.

## The signal's frequency, the sampling frequency and the nyquist rate

Spatial sampling-reconstruction procedures require to take prior decisions on the **spatial resolution** of the sampling tool and on the **spatial sampling frequency**. Spatial resolution is intrinsic to the sampling instrument; on the contrary, the spatial sampling rate is fully manipulable by the experimenter, depending on the spatial distance between consecutive sampling points. How does an experimenter choose an appropriate sampling frequency? The well-known Shannon-Nyquist theorem states that a signal can be reconstructed from its samples, if the original signal has no frequencies above 1/2 the sampling frequency, the so-called Nyquist rate (Nyquist, 1928; Shannon, 1949). Violating this limit exposes to the risk of aliasing, therefore jeopardizing the reconstruction process. Native images are analogic and usually contain the most diverse (virtually all) spatial frequencies and therefore the choice of an appropriate sampling frequency may be impossible (Jerri, 1977). To solve this issue, in sampling-reconstruction procedures the native images are low-pass pre-filtered to form a band-limited signal (Yadav, 2009). Band-passing the native signal is a strategy that minimizes aliasing in favor of blurring. Once the signal has been band-limited to the frequencies of interest, the ideal sampling frequency is automatically identified by the Shannon-Nyquist principle to the double of the low-pass filter.

## Spatial sampling with focal TMS: the need for dense sampling

The **spatial resolution** of TMS depends on the volume of cortex that is stimulated, which depends largely from stimulus intensity, a parameter that varies in a very narrow range in most TMS-brain mapping procedures. We can therefore assume a fixed spatial resolution of TMS of 1–2 cm^2^ (Deng et al., 2013). On the contrary, the **spatial sampling frequency** depends on the spatial distance between two consecutive sampling points. The spatial sampling resolution in 2D images (as is the case of the cortical surface) is commonly expressed in units per distance in a single row of a raster (for example “dots per inch” or dpi) in the case of TMS I will talk about TMS spots per cm. For example, stimulating a spot on the left hemisphere and a control spot on the right hemisphere implies a spatial sampling frequency of 1/15 spots/cm. Let us make a fictive example. A TMS experiment shows that stimulation over the left angular gyrus, compared to sham TMS, produces a loss of accuracy in the capacity to tell apples from oranges. The spatial sampling is a single point and the reconstructed image will be a monomorphic map where the whole cortex represents uniformly the difference between apples and oranges. If a second TMS spot is added in the right angular gyrus, and it shows not to affect apple-orange distinction, the resulting reconstructed map will show a cortical surface split into two exact halves, exactly like and image reconstructed based on two pixels, in one half of which will be represented the apple-orange distinction. I propose that to fulfill the cartographic ambitions of TMS as a brain mapping tool, it should be used with dense spatial sampling, to produce adequate reconstructed maps of brain function. For obvious practical reasons, TMS cannot be a whole-brain measure and dense sampling can be limited to small cortical regions of interest, as has been done in the small number of examples in the literature as for example in (Ellison et al., 2004; Oliver et al., 2009; Stoeckel et al., 2009; Thielscher and Wichmann, 2009; Salatino et al., 2014; Maule et al., 2015; Schaeffner and Welchman, 2017).

## Choosing a behavioral task is an operation of band-filtering the spatial signal of brain functions

**Figure 1:**
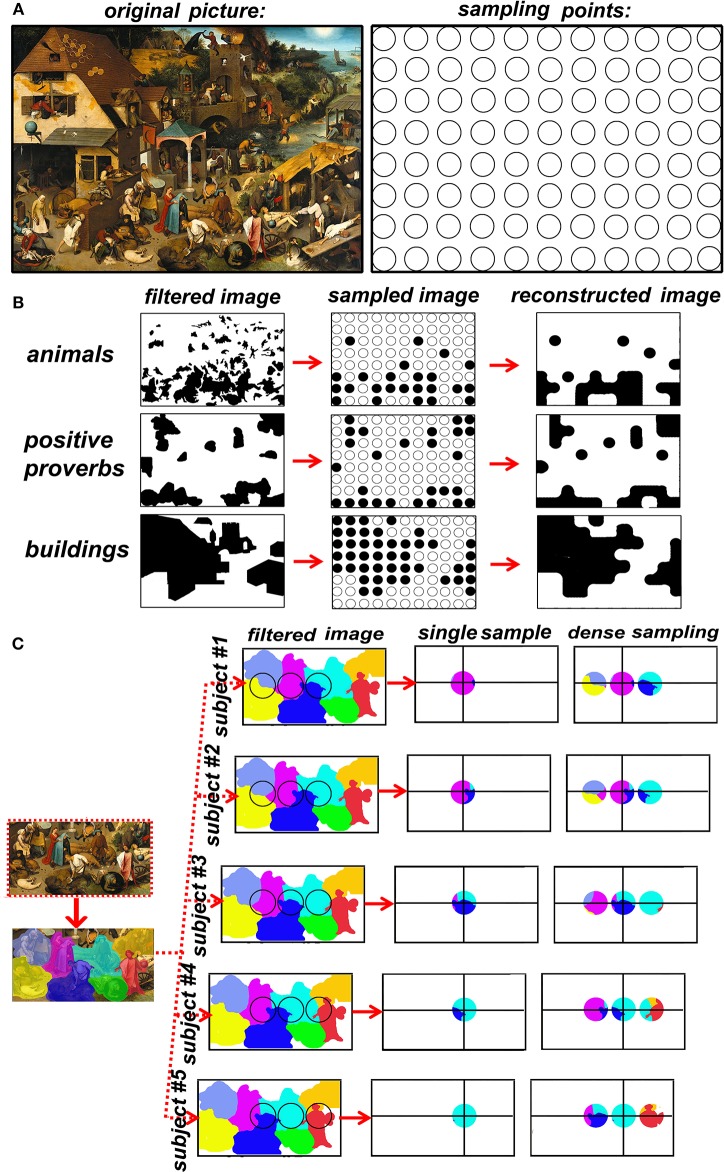
**(A)** Left: Pieter Bruegel the elder's “Netherlandish proverbs” (1559)^1^ and the sampling grid. The image is a faithful photographic reproduction of a two-dimensional, public domain, work of art. **(B)** the effects of the choice of a feature of interest on spatial frequency of the raw signal. **(C)** the advantage of dense sampling in coping with inter-individual variability.

^1^

Once established that spatial sampling with TMS requires dense grids of TMS spots, a spontaneous question arises: how dense is dense? In other words, what is the adequate spatial sampling frequency? We know there is a maximal sampling frequency corresponding to around 1.5 spot/cm, that is dictated by spatial resolution that is inherent to TMS, but the highest sampling frequency I not necessary more efficient. In fact, there is no correct answer, it depends on the maximal signal's spatial frequency. The brain, up to the level of single cortical columns, is rich in spatial frequencies so high that it can be assimilated for our purposes to an analogic image. It is impossible to use TMS sampling frequencies adequate to the fine granularity of brain functions. The solution to this apparently unsolvable problem is band-limiting the signal. Let us get some help on this discussion on spatial sampling from an extraordinarily elaborate analogic picture, Pieter Bruegel the elder's “Netherlandish proverbs” (Figure 1A, left), that I will use as an analogy of the brain surface that we want to map. A hypothetical grid of the painting's coverage at a given sampling frequency is represented in Figure 1A, right. Remember once more that TMS does not produce data by itself, we must therefore choose what characteristics of the signal (painting) to sample. Figure 1B represents 3 examples of sampling choices: *animals* (including humans), *proverbs with positive meaning* (such as “to have the roof tiled with tarts”, versus negative proverbs such as “to be a pillar-biter”) and *buildings*. It is visually immediate that the three categories of interest have a different spatial frequency in the native space (left row). The spatial sampling (middle row) is then used to reconstruct the image (right row). The goodness of the sample-and-reconstruct procedure is testified by how similar the reconstructed image is to the raw image. It is evident that the procedure fails in the case of animals but is acceptable in the case of positive proverbs and buildings. However, the original image did not change (the painting). What changed was the category that we decided to sample, the question that was asked at every spot: “is there an animal in this spot?” or “is there a positive proverb in this spot?” or “is there a building in this spot.” Let us now switch from the pictorial analogy to the TMS/brain system. TMS does not produce data by itself, the type of data is up to the experimenter's choice and depends strictly on the behavioral measurement. For example, I may choose to record response times (RTs) to a somatosensory stimulus to the left IV finger or to the whole upper limb. In the first case, even the maximal TMS sampling frequency will be probably insufficient for the signal's spatial frequency. In the second case, a 0.5 spots/cm sampling frequency will probably be optimal. Concluding, the spatial frequency of the brain signal is dependent exclusively on the behavioral task that I chose to explore the effects of TMS. Our choice of behavioral measures IS an operation of spatial low-pass filtering.

## Advantages of dense sampling

The main advantage of dense sampling with TMS is the production of reliable spatial information on functional brain maps. Sparse sampling is insufficient for mapping most cognitive functions, but dense sampling remains scarcely used in the cognitive neurosciences (Ellison et al., 2004; Oliver et al., 2009; Stoeckel et al., 2009; Thielscher and Wichmann, 2009; Cattaneo and Barchiesi, 2011; Salatino et al., 2014; Sliwinska et al., 2014; Finocchiaro et al., 2015; Maule et al., 2015; Parmigiani et al., 2015; Schaeffner and Welchman, 2017). On the contrary, dense TMS spatial mapping flourished in fields in which accurate spatial mapping is of paramount importance, namely non-invasive pre-surgical assessment of brain functions. If spatial accuracy has direct implications for individual health, dense TMS mapping has made a profound impact, namely in cortical mapping of the motor cortex and of Broca's area (Könönen et al., 2015; Tarapore et al., 2016a,b). Similarly, dense TMS mapping has found applications also in mapping the motor cortex in stroke patients (Byrnes et al., 2001; Thickbroom et al., 2004). A second, priceless advantage of dense TMS mapping is its use in a relatively hypothesis-independent way. Sparse sampling requires a very strong hypothesis on the spatial location of a single region and potentially leads to circular reasoning: “area x is responsible for behavior y” is BOTH an experimental assumption of coil placement AND a desired result. This risk is minimized by dense sampling. A further, practical advantage of dense sampling is that it overcomes individual anatomical variability. A major source of uncertainty undermines the correspondence between the scalp position and the target, i.e. individual variations in cortical topography. The level of uncertainty changes according to the “neuronavigation” method (Sack et al., 2009) and is lower for individual anatomy-based neuronavigation and functional imaging-guided neuronavigation. In both these procedures, however, target localization is sub-optimal for the following reasons. A) macro-anatomy predicts microstructure and function only in a very limited set of regions (Geyer, 2004). B) Spatial localization of single-subject functional data is optimal for producing TMS targets in areas involved in primary cortical processing of sensory or motor information. On the contrary, when it comes to complex behavior, fMRI localization of a TMS target in a single subject has considerable spatial noise, it has a low test-retest repeatability and therefore is potentially fallacious for TMS neuronavigation (Desmond and Glover, 2002; Lund et al., 2005; Pinel et al., 2007). In other words, no matter how accurately I place the TMS coil on the participant's scalp, the underlying signal will be randomly translated in all directions from subject to subject. Let's turn another time to Bruegel's painting. The left side of Figure 1C shows a detail of the lower part of the Proverbs in which single proverbs have been color-coded. The right part of Figure 1C represents five different subjects in which the underlying space to be sampled has been translated to simulate individual anatomical variability of cortical maps. The results of single (sparse) sampling suffer greatly from underlying spatial variability: in half of the subjects a different proverb is sampled. On the contrary, adding a micro-array of dense sampling allows constant coverage of the target region in all subjects. A further advantage of TMS dense-sampling is that it allows to describe multifocal distributions of single cortical functions, as has been demonstrated for phosphenes in the occipital cortex (Thielscher and Wichmann, 2009). Sparse sampling with TMS assumes a 1:1 relation between behavior and brain regions, or in other words, that a single region produces a single behavior. Dense sampling allows for the definition of mosaics of cortical areas involved in a single task. Uneven sampling has advantages as has been proven theoretically in image processing and practically in cortical sampling (Van De Ruit et al., 2015).

## Disadvantages of dense sampling

We claim that dense sampling can improve within and between-subjects *spatial* signal/noise ratio, but there are important sources of spatial noise that are unavoidable. One main source of inhomogeneity in cortical sampling with TMS is gyrification of the cortex. Spatial anisotropy of the cortical surface produces *spatial* noise at 2 levels. A) cortical folds change the depth of the stimulated tissue, and effectiveness of TMS is strongly depth-dependent (Deng et al., 2013). The result is that the bottom of the sulci is unattained by direct stimulation. B) Cortical folds change the orientation of cortical axons, and effectiveness of TMS is orientation-dependent (Ni et al., 2011). It could therefore be that, even at equal depths, two differently-oriented sulcal crowns are stimulated unevenly. The most important limitation of dense spatial sampling is that it only tackles *spatial* noise, but there are other sources of noise in experimental measures. For example, the stochastic variability of participant behavioral performance and of the effects of TMS on it is a source of *behavioral* noise, that is independent from the spatial noise. Functional behavioral noise is tackled in cognitive neuroscience by collecting several repeated trials per experimental condition and averaging them, to increase signal/noise ratio. The more trials are repeated within condition, the more noise is zeroed in favor of signal. However the number of trials is limited by practical, ethical and safety reasons (Rossi and Hallett, 2009). Increasing the number of stimulation spots by dense sampling necessarily increases the number of experimental conditions. However, if experiment duration is limited, this implies that the number of trial repetitions per condition decreases, potentially lowering the behavioral signal/noise ratio. Proper use of dense sampling TMS should imply a tradeoff between the disadvantage of decreased *behavioral* signal/noise ratio and the advantage of increased *spatial* signal/noise ratio. This can be achieved for example by adopting small spatial grids that, however, cover comprehensively a cortical region of interest.

## Author contributions

The author confirms being the sole contributor of this work and approved it for publication.

### Conflict of interest statement

The author declares that the research was conducted in the absence of any commercial or financial relationships that could be construed as a potential conflict of interest.

## Footnotes

^1^https://commons.wikimedia.org/wiki/File:Bruegel_Proverbs.jpg
